# Outcome of staged buccal mucosal graft for repair of long segment anterior urethral stricture

**DOI:** 10.1186/s12894-019-0466-4

**Published:** 2019-05-16

**Authors:** Mohamed Selim, Shady Salem, Eid Elsherif, Atef Badawy, Mohamed Elshazely, Maher Gawish

**Affiliations:** 1 Department of Urology, Faculty of Medicine, Menoufia University, Governorate, Menoufia, Egypt; 2 Department of Urology, Faculty of Medicine, Alazhar University, Governorate, Assiut, Egypt

**Keywords:** Urethra, Stricture, Anterior, Staged

## Abstract

**Background:**

Long anterior urethral stricture due to variable etiological factors constitutes a challenge for reconstruction.

We evaluated our centers experience with cases of long anterior urethral stricture due to different etiologies that were managed by 2-stage substitution urethroplasty using buccal mucosal graft procedure.

**Methods:**

During the period between November 2009 and November 2016. All cases with long anterior urethral stricture that were planned for substitution urethroplasty in our department were enrolled in this study. The first stage was excision of most fibrotic areas of the urethral plate, the remaining of the urethra is laid open and augmented with buccal mucosal graft for second stage closure after 6–9 months.

**Results:**

The study included 123 patients who underwent first stage, 105 patients of them underwent second stage urethroplasty. Eighteen cases were missed after first stage. The mean (range) age was 38.4 (17–60 years). The mean (range) stricture length was 8.3 (4–13 cm). The cause of stricture was idiopathic in 47, inflammatory in 15, lichen sclerosus in 26 and post failed hypospadias repair in 35 patients. First stage was complicated by graft contracture in 11 (8.9%) patients that needed re-grafting, 5(4.1%) patient had bleeding from the buccal mucosa site that needed haemostatic sutures, oral numbness was reported in 7 (5.7%) patients. Second stage was complicated by wound dehiscence in 2(1.9%) patients, restricture in 11 (10.5%), fistula in 6 (5.7%) patients, meatal stenosis in 3 (2.9%). The overall success rate was 79.1% (83 cases out of 105) with a mean (range) follow-up of 34.7 (10–58 months).

**Conclusions:**

Staged urethroplasty using buccal mucosal graft procedure is an effective surgical option for patients with long anterior urethral strictures especially for patients with lichen sclerosus and those with failed previous surgical repair.

## Background

Urethral stricture is a process of progressing spongiofibrosis that leads to subsequent urethral narrowing and persistent lower urinary tract symptoms (LUTS) [1]. The management of long anterior urethral stricture remains an issue of controversy and continuing challenge, especially if it is lengthy or recurrent [2]. The treatment options has been modified form visual internal urethrotomy (VIU) and dilatation to urethroplasty, either anastomotic or augmentation, using buccal mucosal grafts or penile skin flaps [3–5].

The widespread application of buccal mucosal graft is partly due to its favourable characteristics, including a thick epithelium, highly vascularized lamina propria, availability, strength and resistance to infection [6].

Single stage augmentation urethroplasty is recommended for most of penile urethral stricture. Penile skin flaps was the main modality of management of penile urethral strictures [7, 8].

Barbagli and colleagues reported the dorsal free graft urethroplasty initially using skin, but subsequently buccal mucosa, which was a modification of the Monseur technique [9, 10]. Asopa and colleagues described a ventral sagittal urethrotomy approach and dorsally placed BM graft with good success rates [11]. Kulkarni and Barbagli used a technique to preserve the nerve and blood supply to the urethra by one sided dissection of anterior dorsal aspect of the bulbospongiosus muscle [12]. Two-staged reconstruction should be utilized without hesitation on cases of prior failed hypospadias management or those with severe urethral LS [13, 14].

Kulkarni and Barbagli, have suggested that it may be possible to do a one stage procedure with buccal mucosa in patients with LS where there is an adequate roof strip 20F [15]. It is of note though, looking at this series of patients a relatively small number of patients out of the total with LS were suitable for this approach.

Other reasons for the staged procedure are in recurrent complicated cases after previous failed procedures. The two stage skin reconstruction is very appropriate for patients with failed hypospadias, but in case of LS, skin should be avoided and buccal mucosa substitution is recommended. There is a small literature researches relating to this [16] and it is recognized that there is a 22.5% revision for the first stage urethroplasty [17]. These authors concluded that two stage urethroplasty of complex penile urethral stricture had lower restricture rate than one stage, and clearly there is a significant rate of revision of the first (22.5%).

Andrich and Mundy reported that the presence of LS complicating the urethral stricture compromises the rate of success of one stage approach, also there is tendency of recurrence in the marsupialised urethra and dictated perineal urethrostomy as an option for panurethral stricture and elder patients [18].

In this study, we report our experience of the feasibility of staged urethroplasty using buccal mucosal grafting to repair all long penile urethral strictures. We tried to identify the predictors of success. We analysed outcome and complications in relation to the site of stricture (bulpopenile versus penile), length of stricture and etiology.

## Methods

This was an observational study conducted over seven years, from November 2009 to November 2016, at Menoufia and Al Azhar University teaching hospitals. Cases with recurrent anterior urethral stricture, who were subjected to augmentation urethroplasty, were evaluated. The study included all patients with long urethral stricture with previous history of failed attempts to repair including several dilatations or internal uerthrotomy or previous urethroplasty and those with obliterative strictures who are on suprapubic catheters also we included cases with susbected LS based on clinical criteria without histological examination and adult patient with previous failed repeated attempts of repair of penile and penoscrotal hypospadias. We excluded all cases with minimal fibrosis and short strictures with patent urethra that are feasible for single stage management.

Detailed urological and medical histories were obtained. History of trauma to the pelvis, the perineum and/or the genitalia, as well as urethral infections and previous urological procedures (dilatation, DVIU, previous surgical repair of urethral stricture or hypospadias) were recorded. General and complete genitourological examinations were done.

All patients had previous urethral instrumentation in the form of several dilatations or internal uerthrotomy. In addition, thirty-one patients underwent previous urethroplasty elsewhere. Forty-seven patients had suprapubic cystostomy for urinary retention. The patients were treated by the two-stage augmentation urethroplasty using buccal mucosal grafts. All patients signed a detailed formal consent. The study was approved by our institutional ethical committee.

### Preoperative evaluation

New urine culture done for all cases to detect urinary tract infection. Patients with positive cultures were treated with the proper antibiotic before surgery.

The patients were evaluated regarding stricture etiology, length and site. Preoperative retrograde urethrography, previous surgical or endoscopic treatment, surgical findings, postoperative complications, follow up, outcome and success rate were also recorded.

Uroflowmetry, retrograde urethrography and voiding cystourethrography were performed for all patients. Urethroscopy with semirigid cystoscopy was conducted for every case on the day of surgery to confirm the presence of the stricture before proceeding with urethroplasty and to assess the patency and calibre of the urethra.

### Operative details

All surgical procedures were done under general anaesthesia with nasotracheal intubation. All patients were placed in the lithotomy position. A ring retractor was used for retraction and exposure of perineal approaches and hanging up the penis during penile approaches.

Harvesting the buccal mucosal graft The buccal graft was harvested by the assistant surgeon using the technique described by Barbagli and his colleagues [19].

### Urethroplasty using buccal mucosa graft

The reconstruction of the urethra was done in two stages. The buccal mucosal was engrafted to the neourethra alongside the native urethra that was laid open and observed for 6 to 9 months according to the healing of the graft and absence of fibrosis. Then, the second stage closure of the urethra was done.

### First stage buccal mucosal augmentation urethroplasty

For penile urethral stricture, an incision was made longitudinally and dorsolaterally to the healthy urethra. Areas with extensive spongiofibrosis were excised, leaving the healthy portions that were laid open.

**Fig. 1:**
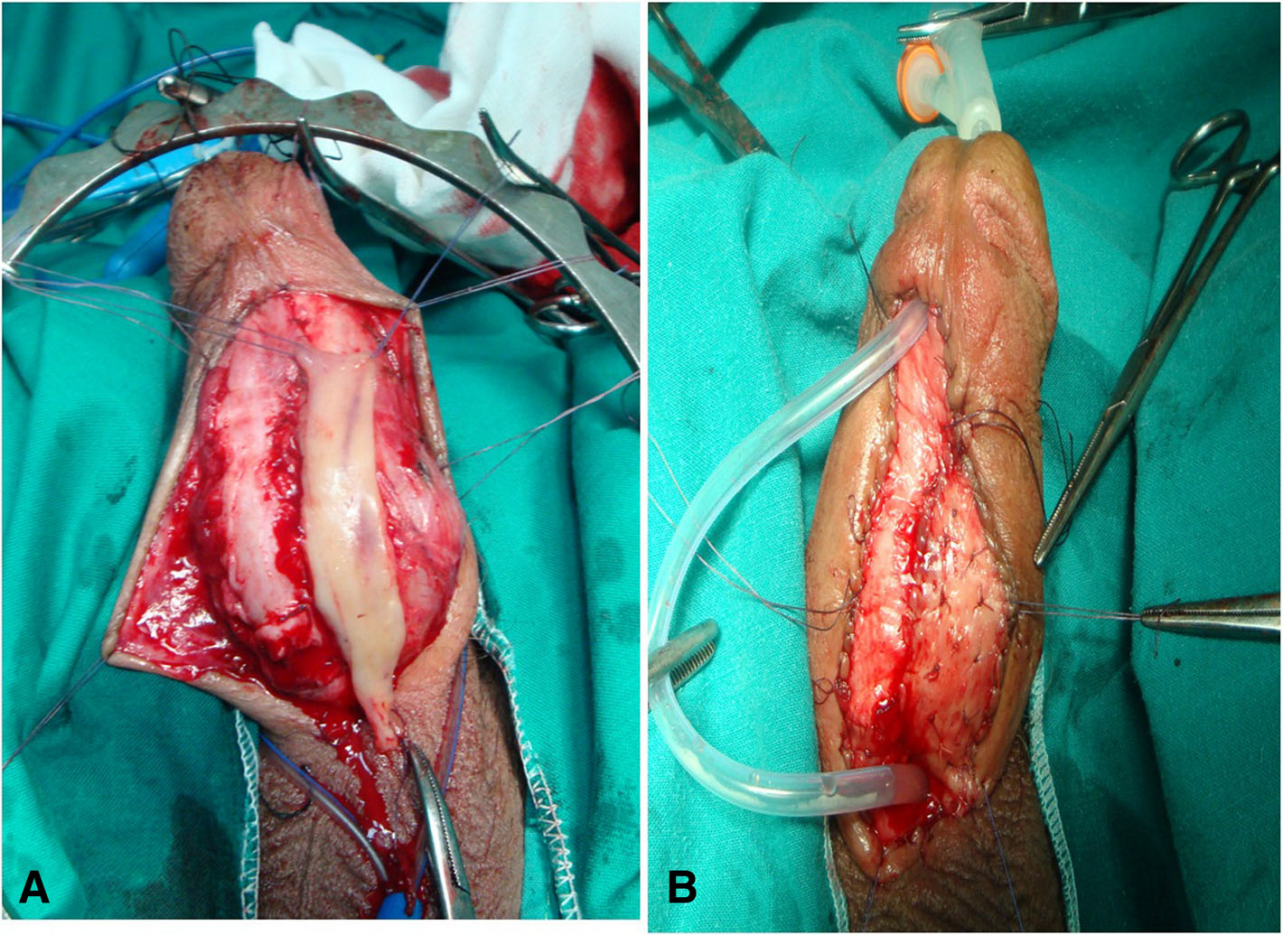
**a**, buccal mucosa graft was tailored and placed beside the marsupialized urethra. 1. **b**, buccal mucosa was fixed alongside the urethra, penile skin, fenestrated, quilted and urethral catheter was fixed

Neourethrostomy of the proximal end of the urethra and marsupialization of the opened urethra were conducted. Buccal mucosal graft was laid to the side of the opened urethra and fixed to it. An incision was made on the edge of the skin, which was then fenestrated and quilted. The 16F silicon urethral catheter was fixed for 5 days (Fig. 1a, b).

For bulbopenile strictures, the incision was made on the ventral of proximal penile shaft and extended through the scrotum with separation of the scrotal compartments to expose the urethra according to the extension of the stricture. The urethra was dissected laterally and then incised dorsolaterally without any circular dissection of the urethra. Some cases with stricture reaching the bulbar urethra beneath bulbospongiosus muscle needed dissection and separation of the muscle at the midline so the dorsolateral incision could be extended to this segment. After that, the urethra was marsupialized and laid open; the buccal mucosal graft was fixed alongside the urethra and quilted. Then, the skin penis and scrotum was fixed along the free side of the graft and the urethra leaving the scrotum splitted around the neomeatus. The dressing was removed 5 days post operative for both penile and bulbopenile stricture and patients were taught how to care for the graft by cleaning the wound with antiseptic solution and application of eye ointment (oxytetracycline HCl) together with vaselinated gauze and continued to do this for 6 weeks to prevent the row areas of the graft from sticking together.

### Second stage urethroplasty

Second stage urethroplasty was preformed 6 to 9 months after the first stage according to the healing process and the absence of fibrosis.

### Operative technique

**Fig. 2:**
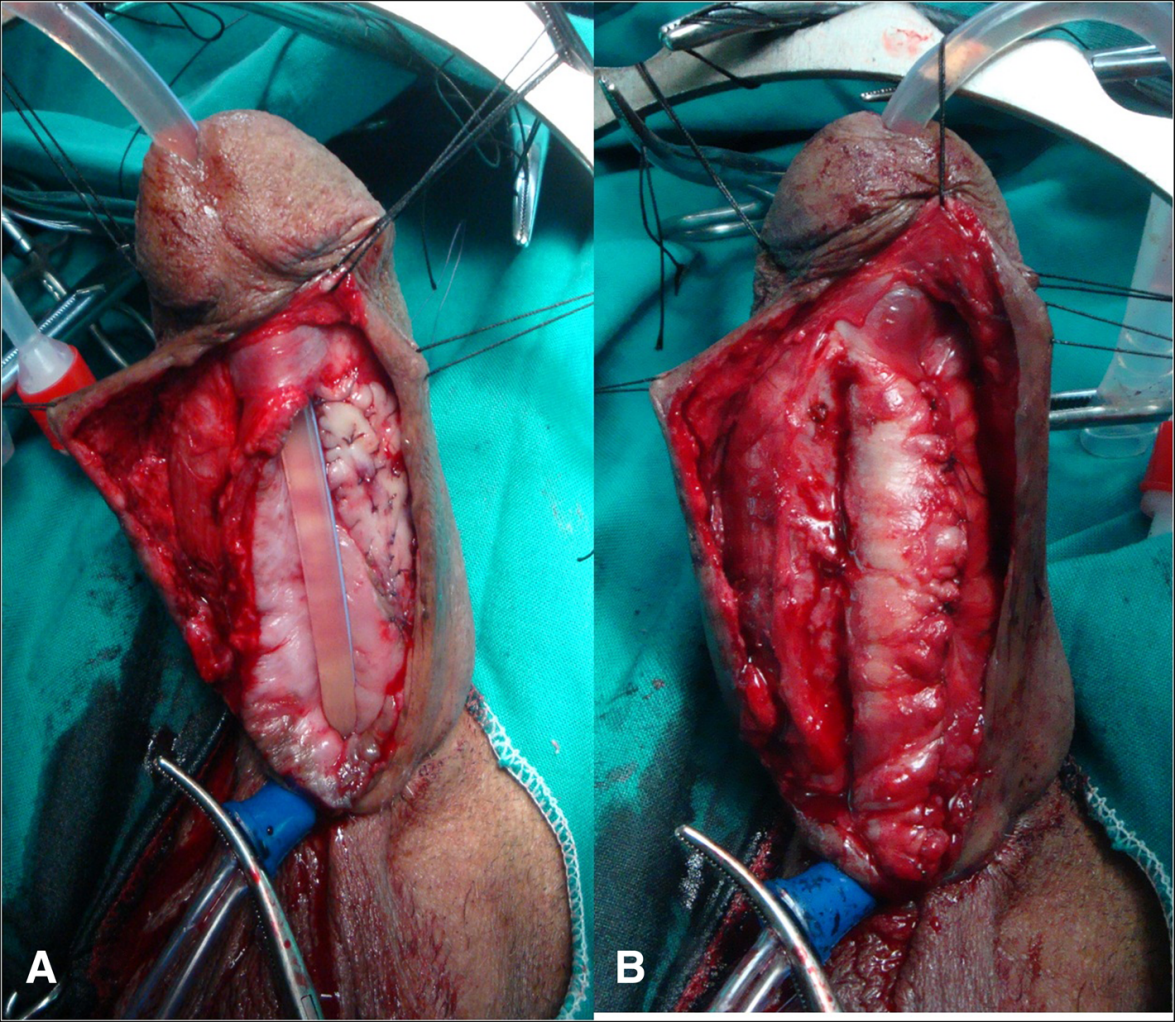
**a**, Roof strip was mobilized by dissection of the skin at the edge of the strip, one stage buccal mucosal roof strip augmentation of the narrow distal neourethra and urethral catheter.2. **b** tubularization of the neourethra

The neourethral tube was outlined and marked giving urethral plate width of 2.5–3 cm. An incision was done and the roof strip was mobilized by dissecting the skin at the edge of the strip (Fig. 2a, b). The urethral tube was closed over 16F silicone urethral catheter.

### Postoperative care

The urethral catheter was left in its place for three weeks and was removed after confirming its healing, by performing peri-catheter urethrogram. Patients were followed up and evaluated regularly till the 6th week from the surgical procedure, then every 3 months in the first year, then every 6 months and then yearly for 5 years. The follow up included clinical observation of the patient, the process of healing of the wound and routine flexible urethrocystoscopy, our defined criteria for success was the patency and prompt healing of the neourethra confirmed by pericatheter urethrogram and absence of restricture on subsequent follow up visits without need for dilatation or endoscopic management.

### Statistical analysis

Data was tabulated and statistically analysed using Stata® software (Stata Corp. 2011. Stata Statistical Software: Release 12. College Station, TX: Stata Corp LP). We used Fisher’s exact test and Mann-Whitney U test, wherever appropriate. We used *P*. value < 0.05 as statistically significant.

## Results

The study included 123 patients who underwent the first stage urethroplasty, 105 patients of them underwent the second stage. Eighteen cases were missed after first stage. The mean (range) age of the patients was 38.4 (17–60 years). The cause of stricture was idiopathic in 47 patients, inflammatory in 15 patients, lichen sclerosus in 26 patients and post failed hypospadias repair in 35 patients. The stricture was in the penile urethra in 68 patients and bulbopenile in 55 patients. The mean (range) length of the overall stricture was 8.3 (4–13 cm), penile stricture was 7.4 (4–10.5 cm) and bulbopenile 9.3 (5–13 cm) respectively with no statistical significance (Table 1).

**Table 1:** Patients’ demography and stricture characteristics (site and length) (*N* = number of cases)

variable	*N* (%)
Total patient, *N*	123
Stricture etiology, *N* (%)	
Idiopathic	47 (38%)
Inflammatory	15 (12%)
Lichen sclerosus	26 (21%)
Post failed hypospadias repair	35 (29%)
Stricture site, *N* (%)	
Bulbopenile	55 (44.7%)
Penile	68 (55.3%)
Length of stricture cm, mean (range)	
Penile	7.4 (4–10.5)
Bulbopenile	9.3 (5–13)

Five patients had mucosal site bleeding that needed hemostatic sutures. Seven patients had oral numbness and were referred to the dentist. Graft contracture was encountered in 11 patients and they needed surgical readjustment before the second stage.

The second stage was done after a mean (range) of 6.7 (6–9 months) after the first stage. Second stage augmentation urethroplasty using buccal mucosa was done for 105 patients. During follow up, early postoperative leakage in the pericatheter urethrogram complicated 9 patients who needed prolonged urethral catheterization up to 4 weeks. Spontaneous healing occurred in 3 patients. Urethrocutaneous fistula developed in 6 patients. Meatal stenosis developed in 3 patients.

Failed cases include wound dehiscence and restricture. Wound dehiscence complicated 2 patients due to wound infection.

Recurrence of stricture occurred in 11 patients. All of these cases had a long history of stricture disease and had previously undergone multiple procedures. Two of them had changes of lichen sclerosus (LS), 4 had multiple failed hypospadias repair attempt and others shared the remaining numbers. The recurrence was distal to the graft site in two patients, multiple in two patients (patchy), and a diffuse narrowing in 7 patients (two penile and 5 bulbopenile).

The overall success rate was 79.1% (83 cases out of 105) with a mean follow-up of 34.7 (10–58 months). (Table 2).

**Table 2:** Complications of 1st and 2nd stage substitution urethroplasty using buccal mucosal graft (*N* = number)

Variable	No
1st stage patients, *N* (%)	123
Mucosal site bleeding	5 (4.1%)
Oral numbness	7 (5.7%)
Graft contracture	11 (8.9%)
Mean time elapsed between 1st and 2nd stage (range) months	6.7 (6–10)
2nd stage patients, *N* (%)	105
Wound dehiscence	2 (1.9%)
Restricture	11 (10.5%)
Fistula	6 (5.7%)
Meatal stenosis	3 (2.7%)
Mean follow-up (range) months	34.7 (10–58)
Failure rate	22 (21%)
Success rate	83 (79%)

Correlating the site of stricture with success rates showed a significantly statistical outcome with higher success rates for penile urethral strictures. Correlating the site with the complications was statistically insignificant. (Table 3).

**Table 3:** Analysis of the site of stricture in relation to outcome and complications (*N* = number)

Variable	Bulbopenile (46)	Penile (59)	*P*. value
Success rate *N* (%)	32 (69.57%)	51 (86.44%)	0.036
Fistula *N* (%)	4 (8.7%)	2 (3.39%)	0.148
Meatal stenosis *N* (%)	1 (2.1%)	2 (3.4%)	0.711
Restricture *N* (%)	7 (15.2%)	4 (6.7%)	0.161
Dehiscence *N* (%)	2 (4.3%)	0 (0%)	0.106

When comparing the size of stricture to the outcome and complications. The results showed statistical significance: the shorter the stricture length, the higher the success rate. The correlation of the size of stricture and complications showed statistical significance with less fistula occurrence and restricture with shorter stricture length. Other complications were insignificant in correlation to the size of stricture (Table 4).

**Table 4:** Analysis of the size of stricture in relation to outcome and complications (N = number)

	*N* (%)	Length of stricture (Mean ± SD)	*P*. value
Outcome			
Successful	83 (79.05%)	7.6 ± 1.7	0.0000
Failed	22 (20.95%)	9.7 ± 1.9	
Fistula formation			
Yes	6 (5.71%)	10.1 ± 1.8	0.0009
No	99 (94.29%)	7.9 ±	
Meatalstenosis			
Yes	3 (2.8%)	8 ± 0.87	0.93
No	102 (97.2%)	8.1 ± 1.99	
Restricture			
Yes	11 (10.4%)	9.8 ± 2.19	0.0018
No	94 (89.6%)	7.89 ± 1.84	
Dehisence			
Yes	2 (1.9%)	9.5 ± .7	0.3113
No	103 (98.1%)	8.1 ± 1.97	

Analysis of outcome in relation to the etiology showed no statistical significance. There was no significant relation between the etiology of stricture and the rate of complication (Table 5).

**Table 5:** Analysis of the etiology of stricture in relation to outcome and complications

	Idiopathic	LS	Failed hypospadias	Inflammatory	total	*P* – value
Outcome						
Successful	37	17	18	11	83	0.06
Failed	5	6	9	2	22	
Fistula						
Yes	2	2	2	0	6	0.8
No	40	20	25	13	98	
Dehiscence						
Yes	0	1	1	0	2	0.7
No	42	22	26	13	103	
Meatal stenosis						
Yes	1	1	1	0	3	0.6
No	41	22	26	13	102	
Restricture						
Yes	2	2	5	2	11	0.5
No	40	21	22	11	94	

## Discussion

The first preliminary report for successful use of buccal mucosa for the urethral reconstruction in adults was in 1992 [20]. El-Kasaby et al., in 1993, reported 90% success rate with buccal mucosa urethroplasty in 20 patients [21]. The buccal mucosa is a preferred substitute of the urethra. It is accustomed to wet condition, resilient to infection, easy to harvest and handle with good take after engraftment [6].

The preferred technique for pendulous urethral stricture reconstruction is urethroplasty using penile skin flap, after the description of the Orandi procedure [7].

The results reported by Wesseles and McAninch for ventral onlay free graft urethroplasty of the pendulous urethra were poor. They did not advocate the use of free graft on penile urethra as they were cautious about the poor vascularity of the corpus spongiosum at the stricture site [22]. This might be valid when placing the graft ventrally on the spongiosum. With this technique, the spongiosum does not contribute significantly to graft support or graft take.

The other concern is detaching a long segment of urethra from the corpora cavernosa, which could compromise its vascularity. However, the distal urethra receives its blood supply in a retrograde way from the glans, which is maintained during dorsal dissection. Also, in dorsal onlay grafting, the graft take depends posteriorly on the corpora cavernosa [23].

Stricture recurrence after bulbar or penile augmentation onlay urethroplasty showed extensive fibrosis involving the whole grafted site and fibrotic ring stricture at proximal or distal ends of the anastomosis with the urethral plate [24, 25].

Reconstructive urologists reported cosmetic problems with pedicled flaps such as scars, webbing and curvature [26].

Two-stage buccal mucosal graft urethroplasty avoids these problems. It has only a scar in the midline of the penis. There is no torsion of the penis due to limited mobilization of the dartos fascia. Also the fixation of the graft to the under lying spongiosum, fixes the neo-urethra and prevents it from “bow-stringing”.

The criteria for successful management was determined, as described by Barbagli et al., as no need for postoperative procedure, including urethral dilation, no urinary retention, no fistula nor chordee [26, 27].

The most common complications following second stage tubularization are urethrocutaneous fistula, dehiscence and recurrent stricture. In our series, the over- all complications rate was 21% (22 cases out of 105 cases). Restricture was the most common complication in our series (11 cases), that could be due prior surgeries with extensive spongiofibrosis that could not be removed totally due deficient of the tissue of ventral aspect of the penis with subsequent incorporation of such segments in the tubularization stage. Urethrocutaneous fistula (6 cases) came in the second place and could be explained due to deficiency of the tunica dartos that were used as second reinforcement layer at the other cases and meatal stenosis (3 cases). This is in agreement with the other series which was reported by Mori and Angermier having complications rate of 19% [14].

Our success rate (79%) after a mean follow up of approximately 3 years (34 months) is comparable to other reported series [13]. Most authors believe that LS adds surgical challenge and increases the chance of restructure [28]. Our results show no significant differences in the overall complications, or failure rate (restricture) between strictures related to LS and those related to other causes. This may be manifest with longer term follow-up beyond 3 years in further reports. Revision rate after repair of complex cases of anterior urethral stricture ranges from 6.7 to 59% as reported by different series [12, 13, 29, 30]. As suggested by Mori and Angermier, to use the term multistage instead of 2 staged urethroplasty [14], we believe that higher success rate could be achieved with further revisions, especially for complex cases.

Limitation of the study is heterogeneity of cases due to different etiologies and being retrospective.

### Conclusions

Staged urethroplasty using buccal mucosal graft is an effective surgical option for patients with long anterior urethral strictures, especially for those with lichen sclerosus or failed previous surgical repair.

## Abbreviations

BXO: Balanitis xerotica obliterans
DVIU: Dilatation and Visual internal urethrotomy
LS: Lichen sclerosus
LUTS: Lower urinary tract symptoms.
VIU: Visual internal urethrotomy
